# A comparative study on the hemodynamic effects of initiating positive airway pressure treatment in patients with obstructive and central sleep apnea

**DOI:** 10.1007/s11325-025-03317-z

**Published:** 2025-04-10

**Authors:** Christoph Müller, Jens Kerl, Dominic Dellweg

**Affiliations:** 1 Department of Internal Medicine, Lahn-Dill-Kliniken, Philipps University of Marburg, Marburg, Germany; 2 Sleep Clinic, Fachkrankenhaus Kloster Grafschaft, Schmallenberg, Germany; 3 Department of Pulmonology, Pius-Hospital Carl von Ossietzky University of Oldenburg, Oldenburg, Germany; 4 Philipps University of Marburg, Baldinger Straße, 35037 Marburg, Germany

**Keywords:** Obstructive sleep apnea, Central sleep apnea, Positive airway pressure, Hemodynamic monitoring, Impedance cardiography

## Abstract

**Purpose:**

Both obstructive (OSA) and central sleep apnea (CSA) are associated with considerable cardiovascular morbidity which argues for treatment initiation with a positive airway pressure (PAP) device even in the absence of significant day-time sleepiness. While the long-term consequences of PAP treatment in patients with sleep disordered breathing have been investigated in several studies, less is known about the immediate hemodynamic effects. Therefore, the present study intended to investigate the immediate effect of PAP treatment on non-invasively measured hemodynamic parameters in 10 patients with either OSAS or CSA.

**Methods:**

During diagnostic and therapeutic conditions, the routine polysomnographic assessment was extended with an impedance cardiography (ICG) system. Statistical analysis was performed to find differences between both groups and conditions. In addition, the relationship between the treatment associated effect on stroke volume (SV) with biometric, polysomnographic, and cardiovascular parameters was assessed.

**Results:**

Comparing both subgroups, we found statistically significant differences for biometric, polysomnographic, and cardiovascular parameters. Patients with CSA were older (*p* = 0.0005) and had higher values for diagnostic (*p* = 0.015) and therapeutic (*p* = 0.029) pulse pressure and the pre-ejection period under diagnostic conditions (*p* = 0.031). In contrast to patients with CSA who exhibited a slight increase of SV and derived parameters under therapeutic conditions, a pronounced decrease was observed in patients with OSAS which was statistically significant for the cardiac index (*p* = 0.038).

**Conclusion:**

Our results indicate that patients with OSAS and CSA who are characterized by unique clinical features may show a distinguishable hemodynamic response to PAP treatment that can be measured non-invasively with ICG.

## Background

Sleep apnea can be due to obstructive or central respiratory events which are classified as two distinct entities and are treated with different positive airway pressure (PAP) devices. Obstructive sleep apnea (OSA) is commonly explained by a collapse of the upper airway during inspiration which is due to decreased pharyngeal muscle tone and a comparably elevated extraluminal pressure during sleep [1]. Breathing excursions against a closed airway lead to negative intrapleural pressure swings, oxygen desaturations and sympathetic activation causing elevated blood pressure levels and a disturbed sleep quality [2–4]. Although there is currently a wide range of available treatment options including surgical approaches, oral appliances or tongue pacemakers [5, 6], PAP treatment with a continuous or adaptive pressure device remains the therapeutic mainstay in OSAS.

Central sleep apnea (CSA) can present in different clinical contexts including chronic heart failure, opioid intake, neurological disorders or as an adaptive state during low oxygen supply. Chronic heart failure is associated with pulmonary congestion and an increased sympathetic tone which predisposes to nocturnal hyperventilation [7]. Low cardiac output and circulatory delay result in a disturbed feedback circuit which leads to hypocapnia and a decreased apnoeic threshold, eventually causing a waxing and waning breathing pattern, called Cheynes-Stokes respiration. Besides treating the underlying condition, CSA is commonly addressed with adaptive servoventilation (ASV) which anticipates respiratory events by predicting the future tidal volume and proving ventilatory support if needed. The device can be set to auto-titration mode or work on two modifiable exspiratory and inspiratory pressure levels. Despite being highly efficient in preventing central respiratory events, ASV is contraindicated in patients with advanced heart failure being associated with an increased mortality among these patients [8].

Although OSA and CSA show a distinguishable pathogenesis, both are related to an increased risk for the development of cardiovascular pathologies like endothelial dysfunction, secondary hypertension, atrial fibrillation or stroke [9–12]. Comorbidities resulting from sleep disordered breathing are explained multifactorially involving intrathoracic pressure changes, apnea induced arousals and disturbances of the normal sleep architecture. Treatment with a positive airway pressure (PAP) device can reverse apnea related pathologies, but may have a potential for hemodynamic impairment which would remain undetected during routine sleep medicine practice. To investigate the immediate cardiovascular effects of PAP treatment in patients with CSA and OSAS, we extended our routine polysomnographic measurements with non-invasive hemodynamic monitoring using impedance cardiography (ICG). ICG strongly correlated with the reference method of thermodilution [13, 14] and had a high re-test reliability [15]. Unlike other techniques like echocardiography or thermodilution, ICG provides a continuous measurement without requiring catheterization. The present study intends to investigate the immediate hemodynamic effects of PAP treatment and tries to find differences in the therapeutic response between patients with CSA and OSAS.

## Methods

### Participants

To investigate the hemodynamic response of PAP treatment, we included 10 patients with CSA related to chronic heart failure (7 patients with preserved and 3 patients with mid-range reduced ejection fraction) and compared them to respiratory distress index (RDI) matched patients with OSAS. Inclusion criteria were a pathological home sleep study with either predominantly central or obstructive events and the indication for diagnostic polysomnography. Patients were excluded if they met any of the following criteria: age younger than 18 years, height below 150 cm or above 210 cm, weight less than 50 kg or greater than 150 kg. All included patients underwent both diagnostic and therapeutic measurements.

### Protocol

Polysomnographic assessment was performed during two consecutive nights between 10 pm and 6 am. If diagnostic polysomnography revealed a pathologically elevated RDI, treatment was initiated with a PAP device. Patients with OSAS were treated with automated positive airway pressure (APAP) (AirSense 10 AutoSet™, ResMed, San Diego, CA, USA), while patients with CSA received treatment with ASV (AirCurve 10 CS Pacewave™, ResMed, San Diego, CA, USA). Both devices were set to auto-titration mode and data were gathered during both nights at a sampling rate of 1 Hz independent of the applied pressure level. Data sets of the impedance cardiograph were assessed and corrected for outliers that may have been caused by artifacts. Informed written consent was given by all participants. The study received ethical approval by the ethics committee of Marburg University and is listed in the German clinical trial register (DRKS).

### Data collection

During two nights under both diagnostic and therapeutic conditions, the SOMNOscreen™ polysomnography system (SOMNOmedics GmbH, Randersacker, Germany) and the CardioScreen^®^ 1000 impedance cardiograph (Medis, Illmenau, Germany) were applied. Airflow measurement was performed with a thermistor and a nasal pressure transducer during the diagnostic night and with the pneumotachograph of the PAP device under treatment conditions. A respiratory induction plethysmograph was used to monitor thoracoabdominal breathing excursions. Continuous monitoring of the systemic blood pressure was performed using the pulse transit technique [16]. Polysomnographic events were scored according to the American Association of Sleep Medicine guidelines [17]. An obstructive apnea is defined as a decrease of the thermal flow signal by ≥ 90% for at least 10 s accompanied by a continued or increased inspiratory effort. An obstructive hypopnea is scored if a decrease of the flow signal by ≥ 30% for at least 10 s which occurs with a drop of the oxygen saturation by at ≥ 4%. Central apneas are distinguished from obstructive events by an absence of thoracoabdominal breathing excursions.

**Fig. 1:**
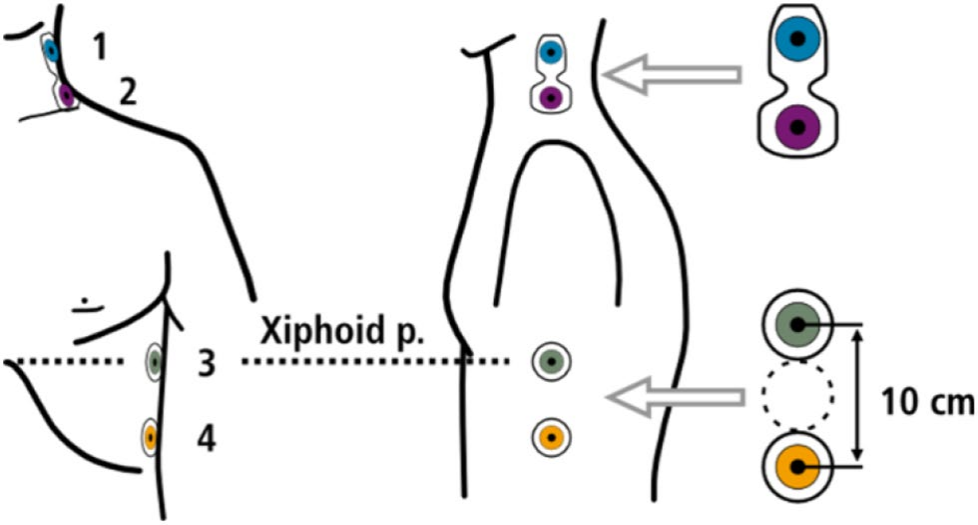
Electrode placement of the impedance cardiograph device

**Fig. 2:**
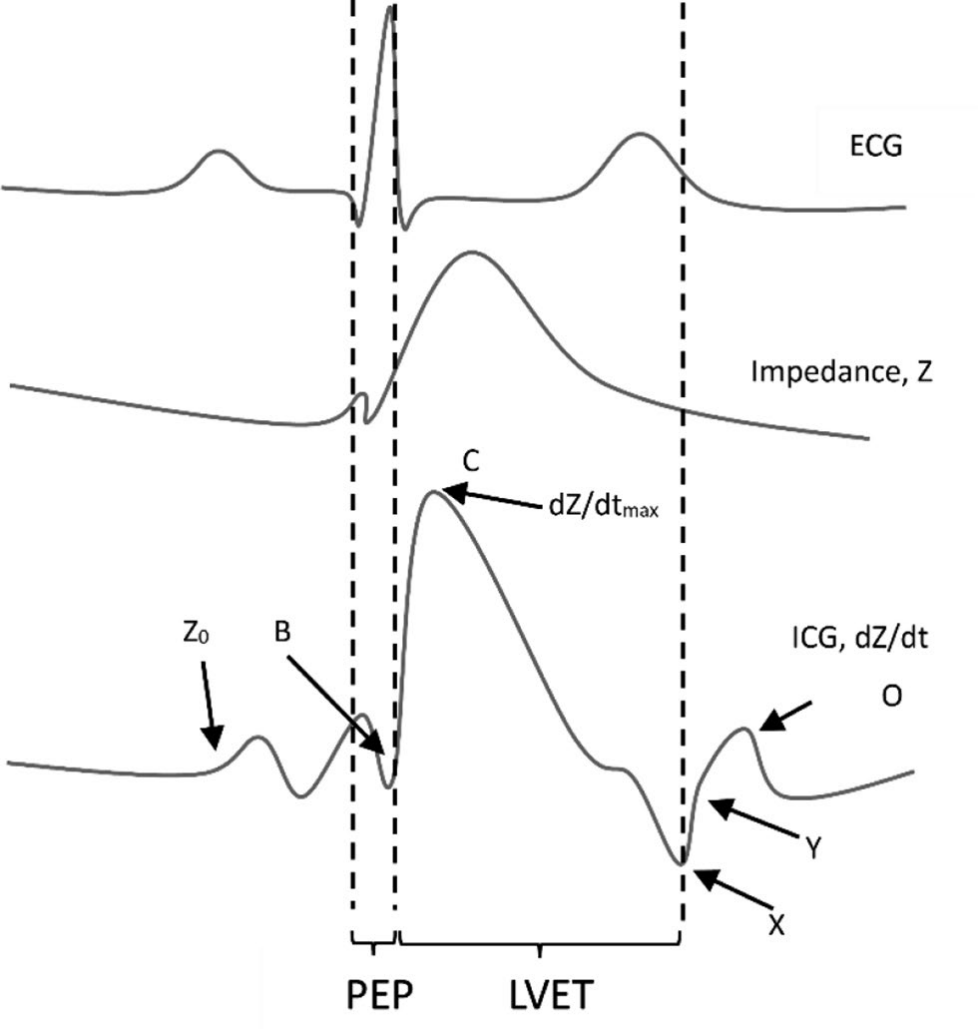
Time course of the electro- and impedance cardiogram with its characteristic turning points and the derived systolic time intervals. Abbreviations: B, opening of the aortic valve; C (dZ/dt_max_), maximal systolic blood flow velocity; dZ/dt, maximal change in impedance; ECG, electrocardiogram; ICG, impedance cardiography; LVET, left-ventricular ejection time; O, closure of the mitral valve; PEP, pre-ejection period; X, closure of the aortic valve; Y, closure of the pulmonic valve, Z_0_, baseline impedance

Hemodynamic monitoring was performed with the impedance cardiograph. Two electrodes connected to two dual sensors were placed on the patients´ lateral side of the neck and along the mid-axillary line of the left chest wall (Fig. 1). The outer sensors create a low amplitude alternating current, which is detected by the inner sensors to measure the change in thoracic impedance over time. To enhance the precision of the measurement of cardiac cycle intervals, an additional pulsoximetry sensor was attached to one ear lobe for recording of pulse volume curves by infrared light. Based on the blood flow dependent changes of the thoracic impedance and electrocardiographic time intervals (Fig. 2), the software “Cardiovascular Lab” calculates different hemodynamic parameters including SV, stroke volume index (SVI), cardiac output (CO), cardiac index (CI), pre-ejection period (PEP), left ventricular ejection time (LVET), ejection fraction (EF), and systolic time ratio (STR).

### Statistical analysis

The acquired data were gathered in EXCEL and analysed using the software XLSTAT© (Addinsoft Lumivero, New York, USA). To search for differences between diagnostic and therapeutic conditions, either a paired t-test or a Wilcoxon-rank test were conducted, after testing for normal distribution with the Shapiro-Wilk test. Mean values of both subgroups were compared with the unpaired t-test or the Mann-Whitney U test. The association between the treatment related change in SV and biometric, polysomnographic, and hemodynamic data was investigated by calculating the Pearson´s correlation coefficient or the Spearman´s rank correlation coefficient. Statistical significance was assumed for a p-value ≤ 0.05.

## Results

### Biometric data

**Fig. 3:**
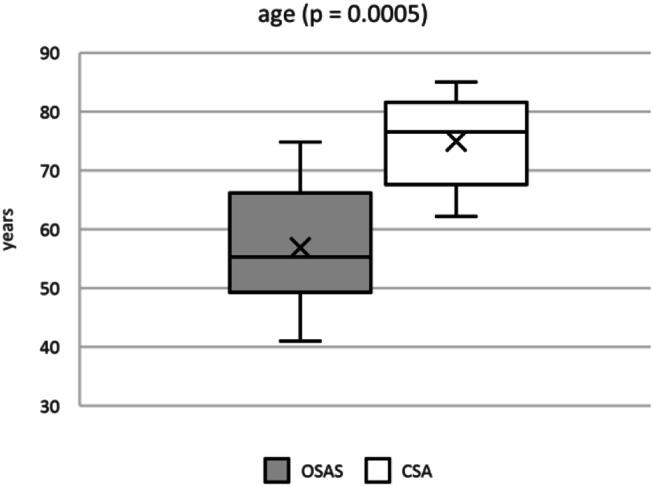
Statistically significant different age of patients with OSAS and CSA. Abbreviations. CSA, central sleep apnea; OSAS, obstructive sleep apnea syndrome

On average participants in both groups were predominantly male and overweight (Table 1). In both groups a pathologically elevated body mass index (BMI) was observed with OSAS patients having a non-statistically significant higher BMI of 34.26 ± kg*m^− 2^ compared to patients with CSA (32.27 ± kg*m^− 2^, *p* = 0.547). The subgroups differed regarding their mean age with CSA patients (74.95 ± 7.56 years) being statistically significant older than patients with OSAS (56.89 ± 11.01 years, *p* = 0.0005) as shown in Fig. 3.

### Polysomnographic parameters

Treatment with a PAP device proved to be efficient in reducing the respiratory distress index (RDI), the occurrence of obstructive apnea (OA) and hypopnea and in lowering the oxygen desaturation index (ODI) for both subgroups (Table 2). Central respiratory events were significantly reduced in patients with CSA (ΔCA -73.94 ± 62.74 h^− 1^, *p* ≤ 0.001). While a statistically significant reduction of the arousal-index was only observed in patients with CSA (ΔArousal-Index − 30.25 ± 99.28%, *p* ≤ 0.05), an effect on the number of counted sleep cycles under treatment conditions did only occur in patients with OSAS (ΔSC 125.00 ± 100.00%, *p* ≤ 0.001). Comparing both groups, statistically significant differences under diagnostic conditions were observed for the occurrence of central respiratory events (1.88 ± 2.07 h^− 1^ vs. 18.43 ± 18,79 h^− 1^, *p* = 0.013), hypopnea (27.35 ± 11.37 h^− 1^, 14.84 ± 14.31 h^− 1^, *p* = 0.044) and RERA (0.37 ± 0.39 h^− 1^ vs. 0.06 ± 0.19 h^− 1^, *p* = 0.010). Under therapeutic conditions, a statistically significant difference between both groups was only observed for OA (1.19 ± 1.39 vs. 0.15 ± 0.25, *p* = 0.010). Treatment with either APAP or ASV led to a statistically significant change in the occurrence of central respiratory events (224.39 ± 395.87% vs. -73.94 ± 62.74%, *p* = 0.0003).

### Hemodynamic parameters

An overview of the hemodynamic data for both subgroups is given in Table 3. Overall, a trend towards a decrease of SV and derived parameters which was statistically significant for CI (ΔCI -6.59 ± 7.32%, *p* = 0.020) was observed in patients with OSAS. In contrast, no significant changes for SV and its related indices were seen in patients with CSA. For both groups, a trendwise significant increase of the PEP under therapeutic conditions and a statistically significant difference under diagnostic conditions was observed (105.67 ± 10.41 ms vs. 122.04 ± 19.50 ms, *p* = 0.031). Patients with CSA showed more elevated systolic blood pressure (SBP) levels with a statistically significant higher pulse pressure under both diagnostic (36.87 ± 9.70 mmHg vs. 59.69 ± 24.92 mmHg, *p* = 0.015) and treatment (38.99 ± 8.60 mmHg vs. 56.32 ± 21.40 mmHg, *p* = 0.029) conditions compared to patients with OSAS.

**Fig. 4:**
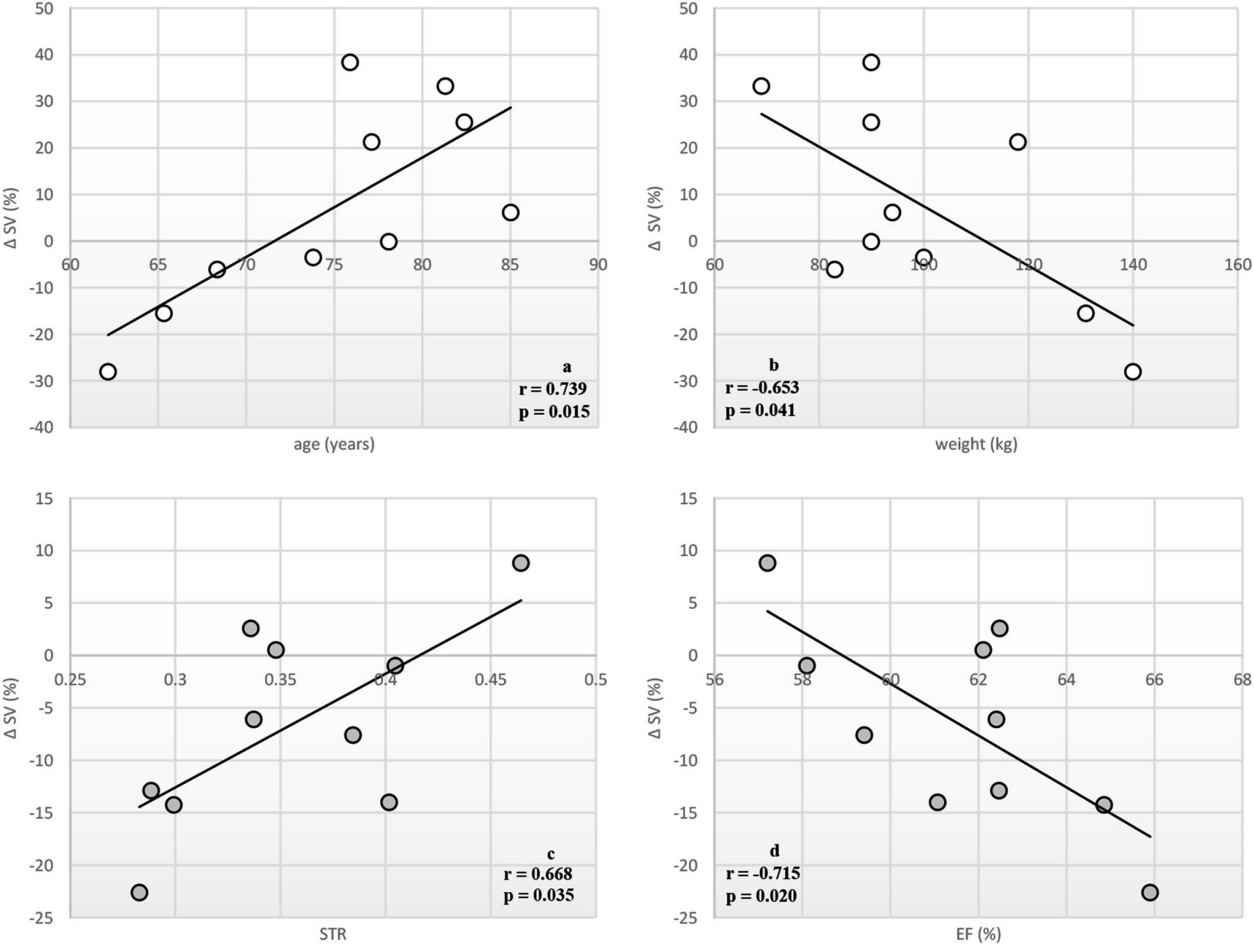
**a.** Correlation between age and relative change in SV in patients with CSA. Figure **b.** Correlation between body weight and relative change in SV in patients with CSA. Figure **c.** Correlation between diagnostic STR and relative change in SV in patients with OSAS. Figure **d.** Correlation between diagnostic EF and relative change in SV in patients with OSAS. Abbreviations. CSA, central sleep apnea; EF, ejection fraction; OSAS, obstructive sleep apnea; STR, systolic time ratio, SV, stroke volume

To assess the relationship between biometric, polysomnographic, and hemodynamic parameters with the relative change in SV, a correlational analysis was conducted for both subgroups. Regarding biometric data, a statistically significant association between patient age (*r* = 0.739, *p* = 0.015, Fig. 4a) and a negative relationship with patient weight (*r*=-0.653, *p* = 0.041, Fig. 4b) was observed for the subgroups with CSA. A positive correlation between the arousal-index under therapeutic conditions and the change in SV was seen for patients with CSA (*r* = 0.626, *p* = 0.071). Patients with OSAS exhibited a trendwise negative relationship between the change in counted sleep cycles (*r*=-0.577, *p* = 0.081) with the change in SV under treatment conditions. A trendwise negative correlation with the change in HR (*r*=-0.515, *p* = 0.096) and the STR (*r*=-0.607, *p* = 0.063) was shown. For patients with OSAS, a negative relationship with the EF (*r*=-0.715, *p* = 0.020, Fig. 4c) and a positive relationship between both the PEP (*r* = 0.624, *p* = 0.054) and the STR (*r* = 0.668, *p* = 0.035, Fig. 4d) were observed.

## Discussion

In the present study, we could demonstrate the immediate hemodynamic effects of treatment initiation with a PAP device in patients with OSAS and CSA by applying ICG as a continuous hemodynamic monitoring technique. On polysomnography, the treatment efficacy of APAP and ASV were approved which both led to a decrease of obstructive and central respiratory events and related parameters like the ODI and the arousal-index.

Comparing both subgroups, patients with CSA were significantly older which confirms epidemiological studies reporting a mean age within the seventh and eighth decade of life [8]. Our study groups were also representative with regards to the predominance of male participants and an increased body weight. Relating to the pathophysiology of both entities, an advanced age in patients with CSA would partly be explained by the increasing incidence of chronic heart failure with concomitant CSA. Some authors conceptualize sleep disordered breathing on a pathophysiological spectrum beginning with OSAS as part of a metabolic syndrome which progresses to CSA as diastolic dysfunction and chronic heart failure become increasingly more prevalent [18].

Both OSAS and CSA are associated with increased sympathetic activation as a result of nocturnal arousals which lead to systemic hypertension as it was observed in both groups. Interestingly, SBP was more elevated in patients with CSA which was associated with a statistically significant higher PP compared to patients with OSAS. This could either be explained by a stronger sympathetic activation due to central respiratory events or by higher baseline SBP levels. Although sympathetic overactivation during periods of CSA is based on substantial evidence [19], the difference in PP between both subgroups is best explained by the pre-existing cardiovascular status. An increasing arterial stiffness with patient age would cause a higher PP in patients with CSA which would not be immediately reversible with PAP treatment [20, 21]. A treatment related decrease of the systemic blood pressure which was observed in several studies and most commonly related to reduced sympathetic activation [22, 23], could not be reproduced in the study presented. Instead, blood pressure values remained relatively stable during both conditions. However, the PEP which has been referred to as a valid surrogate parameter for cardiac sympathetic nervous system stimulation [24, 25], showed a trendwise statistically significant prolongation under treatment conditions. In addition, the duration of the PEP under diagnostic conditions was significantly shorter in patients with OSAS which could either be explained by a stronger sympathetic activation or by a reduced myocardial contractility in patients with CSA. With regards to biometric data, overweight is not only associated with an increasing likelihood in the occurrence of OSA [10], but would also explain a higher baseline sympathetic activation [26].

Besides these indirect effects of PAP treatment, intrathoracic pressure elevations can have an immediate hemodynamic impact which may depend on the underlying cardiovascular condition.

The presumed reduction of left ventricular pre- and afterload due to increased intrathoracic pressure levels would in sum cause a decreasing SV in the absence of heart failure. This is mainly explained by a decreasing transaortic (transmural) pressure gradient and a drop of the systemic venous return [27]. It should be mentioned that the cardiovascular response to changes in intrathoracic pressure levels may also depend on left ventricular morphology. While a treatment related reduction of the transmural pressure gradient may reduce left ventricular wall stress improving myocardial contractility in patients with eccentric hypertrophy [28], the drop of venous return may lead to hemodynamic impairment in patients with a stronger preload dependency, e.g. in concentric left-ventricular hypertrophy [29].

Most of these assumptions are based on experiments performed in the context of invasive positive pressure ventilation and may not be generalized to non-invasive PAP devices. The small number of studies investigating the immediate hemodynamic effects of PAP treatment is partly due to a lack of hemodynamic monitoring in routine sleep medicine practice. Bioimpedance appears to be most appropriate to monitor hemodynamic parameters during sleep studies since it enables a continuous assessment without the need for catheterization and is well tolerated by most patients.

## Conclusion

In essence, by extending the routine polysomnography with a non-invasive hemodynamic monitoring device, we could demonstrate the effect of PAP treatment on hemodynamic parameters in patients with OSAS and CSA. Both subgroups differed with regards to biometric, polysomnographic and hemodynamic data. Our results approved treatment with ASV and APAP to be efficient in reducing the occurrence of central and obstructive events and indicate that PAP devices may have an immediate effect which can be measured non-invasively with ICG. Therefore, we conclude that non-invasive hemodynamic monitoring may be considered when PAP treatment is initiated in patients at risk of hemodynamic impairment.

**Table 1:** Biometric data of both subgroups

Characteristics	OSAS (*n* = 10)	CSA (*n* = 10)	OSAS vs. CSA (*p*-value)
Age (years)	56.89 ± 11.01	74.95 ± 7.56	0.0005
Gender (female)	3	1	0.264
Height (cm)	176.70 ± 8.97	176.30 ± 10.61	0.623
Weight (kg)	105.90 ± 22.87	100.50 ± 22.28	0.599
BMI (kg*m^− 2^)	34.26 ± 8.09	32.27 ± 6.29	0.547

Abbreviations BMI, body mass index; CSA, central sleep apnea; OSAS, obstructive sleep apnea

**Table 2:** Polysomnographic data of both subgroups

Parameters	OSAS (*n* = 10)	CSA (*n* = 10)	OSAS vs. CSA (*p*-value)
RDI, Dx (h^− 1^)	45.45 ± 18.87	44.39 ± 18.12	0.899
RDI, Tx (h^− 1^)	8.56 ± 5.72	8.53 ± 8.05	0.992
Δ RDI (%)	-80.10 ± 12.94^***^	-81.41 ± 15.83^***^	0.832
OA, Dx (h^− 1^)	12.60 ± 16.95	5.25 ± 9.56	0.147
OA, Tx (h^− 1^)	1.19 ± 1.39	0.15 ± 0.25	0.010
Δ OA (%)	-75.59 ± 29.32^*^	-92.54 ± 15.86^*^	0.328
CA, Dx (h^− 1^)	1.88 ± 2.07	18.43 ± 18.79	0.013
CA, Tx (h^− 1^)	2.64 ± 3.83	1.26 ± 2.46	0.351
Δ CA (%)	224.39 ± 395.87	-73.94 ± 62.74^**^	0.0003
Hypopnea, Dx (h^− 1^)	27.35 ± 11.37	14.84 ± 14.31	0.044
Hypopnea, Tx (h^− 1^)	4.74 ± 3.49	5.16 ± 4.93	0.828
Δ Hypopnea (%)	-76.74 ± 28.49^**^	-50.59 ± 64.26^**^	0.255
RERA, Dx (h^− 1^)	0.37 ± 0.39	0.06 ± 0.19	0.010
RERA, Tx (h^− 1^)	0.51 ± 0.54	0.32 ± 0.70	0.106
Δ RERA (%)	43.33 ± 198.17	100.00 ± 352.77	0.474
ODI, Dx (h^− 1^)	36.96 ± 19.37	35.76 ± 14.31	0.901
ODI, Tx (h^− 1^)	5.56 ± 5.55	9.02 ± 9.50	0.333
Δ ODI (%)	-83.10 ± 13.68^**^	-73.27 ± 30.04^**^	0.321
Arousal-Index, Dx (h^− 1^)	43.73 ± 33.77	38.85 ± 33.13	0.748
Arousal-Index, Tx (h^− 1^)	22.13 ± 22.60	14.67 ± 11.04	0.362
Δ Arousal-Index (%)	-41.06 ± 37.19	-30.25 ± 99.28^*^	0.751
SC, Dx	1.00 ± 0.67	0.80 ± 0.79	0.569
SC, Tx	2.40 ± 0.97	1.56 ± 1.24	0.053
Δ SC (%)	125.00 ± 100.00^**^	50.00 ± 83.67	0.163

Abbreviations AHI, apnea-hypopnea-index; CA, central apnea; CSA, central sleep apnea; Dx; diagnostic; OA, obstructive apnea; ODI, oxygen desaturation index; OSAS, obstructive sleep apnea syndrome; RERA, respiratory event related arousal; RDI, respiratory distress index; SC, sleep cycles, Tx, therapeutic; *** indicating *p* ≤ 0.0001; ** indicating *p* ≤ 0.001; * indicating *p* ≤ 0,05

**Table 3:** Hemodynamic data of both subgroups

Parameters	OSAS (*n* = 10)	CSA (*n* = 10)	OSAS vs. CSA (*p*-value)
HR (bpm), Dx	62.87 ± 9.77	61.03 ± 9.54	0.675
HR (bpm), Tx	62.59 ± 9.87	57.68 ± 9.01	0.260
Δ HR (%)	-0.44 ± 4.64	-4.95 ± 9.64	0.199
SV (ml), Dx	109.12 ± 21.73	98.62 ± 25.52	0.325
SV (ml), Tx	101.13 ± 18.72	102.73 ± 24.31	0.871
Δ SV (%)	-6.66 ± 9.52	7.16 ± 21.82	0.083
SVI (ml), Dx	49.18 ± 7.10	44.42 ± 11.50	0.281
SVI (ml), Tx	45.55 ± 5.25	46.27 ± 10.95	0.852
Δ SVI (%)	-6.66 ± 9.52	7.16 ± 21.82	0.083
CO (ml), Dx	6.84 ± 2.04	6.01 ± 2.05	0.371
CO (ml), Tx	6.38 ± 1.87	5.79 ± 1.16	0.408
Δ CO (%)	-6.59 ± 7.32^*^	3.37 ± 26.73	0.271
CI (ml), Dx	3.05 ± 0.60	2.71 ± 0.92	0.332
CI (ml), Tx	2.84 ± 0.54	2.61 ± 0.52	0.329
Δ CI (%)	-6.59 ± 7.32^*^	3.37 ± 26.73	0.271
EF (%), Dx	61.60 ± 2.75	58.30 ± 4.31	0.199
EF (%), Tx	62.59 ± 9.87	57.85 ± 5.48	0.361
Δ EF (%)	-3.14 ± 3.32	-0.77 ± 6.01	0.289
PEP (ms), Dx	105.67 ± 10.41	122.04 ± 19.50	0.031
PEP (ms), Tx	113.96 ± 10.24	126.08 ± 21.29	0.122
Δ PEP (%)	8.32 ± 10.06	3.78 ± 12.11	0.247
LVET (ms), Dx	302.23 ± 15.05	308.26 ± 29.51	0.572
LVET (ms), Tx	301.93 ± 10.24	314.06 ± 29.29	0.303
Δ LVET (%)	-0.13 ± 4.17	2.06 ± 6.19	0.366
STR, Dx	0.35 ± 0.06	0.40 ± 0.07	0.144
STR, Tx	0.38 ± 0.03	0.41 ± 0.09	0.292
Δ STR (%)	8.13 ± 15.46	2.16 ± 6.19	0.419
SBP (mmHg), Dx	128.38 ± 8.61	140.34 ± 27.42	0.205
SBP (mmHg), Tx	129.80 ± 12.75	141.62 ± 26.14	0.215
Δ SBP (%)	1.17 ± 8.21	2.51 ± 19.94	0.846
DBP (mmHg), Dx	91.50 ± 10.24	80.64 ± 13.09	0.053
DBP (mmHg), Tx	90.81 ± 11.78	85.30 ± 16.45	0.401
Δ DBP (%)	-0.47 ± 9.76	6.08 ± 14.90	0.260
PP (mmHg), Dx	36.87 ± 9.70	59.69 ± 24.92	0.015
PP (mmHg), Tx	38.99 ± 8.60	56.32 ± 21.40	0.029
Δ PP (%)	14.27 ± 49.82	4.15 ± 51.32	0.436

Abbreviations CI, cardiac index; CO, cardiac output; DBP, diastolic blood pressure; Dx, diagnostic; DBP, diastolic blood pressure; EF, ejection fraction; HR, heart rate; LVET, left ventricular ejection time; OSAS; obstructive sleep apnea; PEP, pre-ejection period; PP, pulse pressure; SBP, systolic blood pressure; STR, systolic time ratio; SV, stroke volume; SVI, stroke volume index; Tx, therapeutic

* indicating *p* ≤ 0,05

## Data Availability

The datasets generated during and/or analysed during the current study are available from the corresponding author on reasonable request.

## Abbreviations

APAP: Automatic positive airway pressure
ASV: Adaptive servoventilation
BMI: Body mass index
CI: Cardiac index
CO: Cardiac output
CSA: Central sleep apnea
DBP: Diastolic blood presssure
DRKS: German clinical trials register
ICG: Impedance cardiography
LVET: Left-ventricular ejection time
ODI: Oxygen desaturation index
OR: Odds ratio
OSA: Obstructive sleep apnea
OSAS: Obstructive sleep apnea syndrome
PAP: Positive airway pressure
PEP: Pre-ejection period
RDI: Respiratory distress index
REM: Rapid eye movement
SBP: Systolic blood pressure
STR: Systolic time ratio
SV: Stroke volume
SVI: Stroke volume index
